# Effects of participatory organizational interventions on mental health and work performance: a protocol for systematic review and meta-analysis

**DOI:** 10.1093/joccuh/uiae028

**Published:** 2024-05-28

**Authors:** Mako Iida, Asuka Sakuraya, Kotaro Imamura, Hiroki Asaoka, Hideaki Arima, Emiko Ando, Akiomi Inoue, Reiko Inoue, Mai Iwanaga, Hisashi Eguchi, Yasumasa Otsuka, Yuka Kobayashi, Yu Komase, Kazuto Kuribayashi, Natsu Sasaki, Kanami Tsuno, Ayako Hino, Kazuhiro Watanabe, Takeshi Ebara, Akihito Shimazu, Norito Kawakami, Akizumi Tsutsumi

**Affiliations:** Department of Mental Health, Graduate School of Medicine, The University of Tokyo, Tokyo, Japan; Department of Digital Mental Health, Graduate School of Medicine, The University of Tokyo, Tokyo, Japan; Department of Digital Mental Health, Graduate School of Medicine, The University of Tokyo, Tokyo, Japan; Department of Mental Health, Graduate School of Medicine, The University of Tokyo, Tokyo, Japan; Department of Psychiatry, Shinagawa Ekimae Mental Clinic, Tokyo, Japan; Hori Mental Clinic, Fukushima, Japan; Institutional Research Center, University of Occupational and Environmental Health, Japan, Kitakyushu, Japan; Department of Public Health, Kitasato University School of Medicine, Sagamihara, Japan; Department of Community Mental Health & Law, National Center of Neurology and Psychiatry, National Institute of Mental Health, Tokyo, Japan; Department of Mental Health, Institute of Industrial Ecological Sciences, University of Occupational and Environmental Health, Japan, Kitakyushu, Japan; Institute of Human Sciences, University of Tsukuba, Tokyo, Japan; Department of Clinical Psychology, Faculty of Social Policy & Administration, Hosei University, Tokyo, Japan; Healthcare Development Division, Fujitsu Japan Limited, Tokyo, Japan; Department of Psychiatric Nursing, Faculty of Chiba Nursing, Tokyo Healthcare University, Chiba, Japan; Department of Mental Health, Graduate School of Medicine, The University of Tokyo, Tokyo, Japan; School of Health Innovation, Kanagawa University of Human Services, Kawasaki, Japan; Department of Mental Health, Institute of Industrial Ecological Sciences, University of Occupational and Environmental Health, Japan, Kitakyushu, Japan; Department of Public Health, Kitasato University School of Medicine, Sagamihara, Japan; Department of Ergonomics, Institute of Industrial Ecological Sciences, University of Occupational and Environmental Health, Japan, Kitakyushu, Japan; Faculty of Policy Management, Keio University, Fujisawa, Japan; Department of Digital Mental Health, Graduate School of Medicine, The University of Tokyo, Tokyo, Japan; Department of Public Health, Kitasato University School of Medicine, Sagamihara, Japan

**Keywords:** participatory organizational intervention, workplace, occupational mental health, review, protocol

## Abstract

**Introduction:**

Participatory organizational interventions to improve psychosocial working conditions are important for a safe and healthy work environment. However, there are few systematic reviews or meta-analyses investigating the effects of these interventions on workers’ mental health and work-related outcomes. We intend to apply the protocol for systematic review and meta-analysis to examine the effect of participatory organizational intervention on mental health and work performance.

**Methods and analysis:**

The participants, interventions, comparisons, and outcomes (PICO) of the studies in this systematic review and meta-analysis are defined as follows: (P) inclusion of all workers, (I) participatory organizational intervention, (C) treatment as usual or no intervention (including waitlist control), and (O) mental health and work performance. Published studies will be searched using the following electronic databases: PubMed, Embase, PsycINFO, PsycArticles, and Japan Medical Abstracts Society. Studies that (1) include participatory organizational intervention, (2) include participants who were working as of the baseline survey period, (3) assess mental health or work performance outcomes, (4) use a cluster randomized controlled trials design, (5) are published in English or Japanese, and (6) are published in peer-reviewed journals (including advanced online publication) will be included. Study selection and the risk-of-bias assessment will be performed independently by 2 reviewers. A meta-analysis will be performed to statistically synthesize the included studies. Publication bias will be assessed for meta-bias using Egger's test as well as visually on a funnel plot. We will assess heterogeneity by using the Q statistic.

## Key points

- **What is already known on this topic:** Although several cluster randomized controlled trials (cRCTs) revealed the effect of participatory organizational interventions on enhancing workers’ mental health and work-related outcomes, there are few systematic reviews or meta-analyses that gather evidence from cRCTs on the effects of the intervention on these outcomes.
- **What this study adds:** This is a systematic review and meta-analysis protocol specifically to examine the effect of participatory organizational interventions on mental health and work performance among workers.
- **How this study might affect research, practice, or policy:** This research will demonstrate how this type of intervention can impact the well-being and productivity of workers. Considering the importance of well-being and productivity at a workplace, the findings of this study will be useful for public and occupational health.

## Introduction

Negative work conditions such as a hazardous work environment and work organization, harmful or poor work conditions, or poor work relationships can significantly deteriorate the well-being of workers.^1^ To promote and maintain the highest degree of physical, mental, and social well-being of workers in all occupations, it is essential to guarantee a secure and healthy workplace for all workers.^2–^4 For example, higher subjective social support at work, control over work, skill use, and job variety were associated with greater well-being (greater satisfaction, positive and negative affective balance, or less psychiatric disorder).^5^ On the other hand, high levels of job demands, conflict demands, and effort-reward imbalance were associated with less well-being (less satisfaction or high psychiatric disorder).^5^^,^^6^ For a safe and healthy work environment, it is important to have organizational interventions to improve these psychosocial working conditions.^1^^,^^7–10^ Organizational interventions are “planned actions that primarily directly target working conditions with the aim of promoting and maintaining the highest degree of physical, mental, and social well-being of workers in all occupations.”^11^

Numerous scholars have suggested involving workers in implementing organizational interventions.^10^^,^^12–15^ For instance, the International Organization for Standardization (ISO) 45 003 guideline suggests that the participation of workers is essential for developing, planning, implementing, maintaining, evaluating, and continually improving healthy and safe workplaces and managing psychosocial risk.^15^

Workers’ participation increases their control, sense of fairness, justice, and support; their involvement also helps optimize the intervention’s fit to the organizational culture and context; hence, promoting worker participation is a necessary component of organizational intervention.^10^ Sakuraya et al^11^ defined participatory organizational intervention as “workers participate on steps of an intervention, such as action planning, implementing, evaluating, and reviewing the intervention.” In such participatory organizational interventions, management consent and support are essential.^10^ Several previous studies have set and emphasized the step of management participation in this type of intervention.^16^^,^^17^ Furthermore, the process of workers participating in an organizational intervention with the agreement of the management could correspond to organizational justice. For example, in such participatory organizational interventions, the decision-making process should not be biased by personal self-interest or preconceptions but should reflect the views of various stakeholders, which corresponds to the rules of procedural justice (eg, bias suppression and representativeness).^18^^,^^19^ Also, because workers are treated with respect and care, participatory organizational interventions are also considered to be a state of high interactional justice.^20^ Therefore, such participatory interventions are important management practices that can enhance organizational justice.

Several cluster randomized controlled trials (cRCTs) revealed the effect of participatory organizational interventions. For mental health outcomes, the participatory organizational intervention has effects on improved minor psychiatric morbidity (General Health Questionnaire)^16^ and depersonalization (subscale of burnout).^21^ For work-related outcomes, psychosocial work environment (eg, co-worker support and well-defined and realistic workplace goal),^22^ sickness absence,^23^ and job performance^16^ were also improved by participatory organizational intervention. Thus, participatory organizational intervention may be promising for enhancing mental health and work-related outcomes.

There are few systematic reviews or meta-analyses that gather evidence from cRCTs on the effects of participatory organizational intervention on workers’ mental health and work-related outcomes. In previous systematic reviews, organizational interventions had favorable impacts on mental health (eg, decreased distress or burnout)^7^^,^^9^ and work-related outcomes (e.g., absenteeism, sickness absence).^7^^,^^8^ However, these reviews included nonparticipatory organizational interventions as well as participatory ones. Another systematic review reported a meta-analysis examining the effect of participatory organizational interventions on stress levels (eg, occupational stress or burnout), albeit with nonsignificant results (standardized mean difference [SMD]: −0.12; 95% CI: −0.30 to 0.05).^24^ However, this meta-analysis targeted only health care workers and included only 2 studies. Thus, further systematic reviews and meta-analyses are needed to gain a more comprehensive understanding of the effects of this intervention among general workers.

Therefore, the objective of this study is to examine the effect of participatory organizational intervention on mental health and work performance among all workers. This review will treat outcomes related to workers’ well-being or productivity. To the best of our knowledge, this will be the first systematic review and meta-analysis conducted specifically to investigate the effect of participatory organizational interventions among workers. Especially, our review will include only cRCTs because a non-cRCT study cannot avoid the influence of confounding factors, which cause bias in intervention effect estimates.^25^ Also, in non-cRCT designs, imbalances in participant selection or in dropouts between intervention and control groups are more likely to occur, considering the group assignment may be influenced by a variety of factors, including study participant motivation in each cluster.^25^ Accordingly, the systematic review and meta-analysis, including only cRCTs, is meaningful for high-quality evidence with a low risk of bias regarding the effects of this intervention.

## Methods and analysis

### Study design

This is a systematic review and meta-analysis protocol for cRCT studies, following the Preferred Reporting Items for Systematic Reviews and Meta-Analysis protocols (PRISMA-P) guideline^26^ (Appendix 1). The systematic review and meta-analysis will be reported according to the PRISMA 2020 guideline.^27^ The study protocol was registered at the UMIN registry (registration number: UMIN000049453).

### Eligibility criteria

The participants, interventions, comparisons, and outcomes (PICO) of the studies in this systematic review and meta-analysis are defined as follows: (P) inclusion of all workers, (I) participatory organizational intervention, (C) treatment as usual or no intervention (including waitlist control), and (O) mental health and work performance. The definition of participatory organizational intervention is based on an opinion paper by Sakuraya et al^11^ from 2023. For mental health outcomes, positive mental health (eg, optimism, satisfaction, positive affect, well-being, or work engagement), or mental health conditions (eg, mental disorders, depression, burnout, or stress) will be included. In addition, work performance outcomes, such as work capacity evaluation, effectiveness, or presenteeism, will be included. There will be no exclusion criteria for workers based on their employment status, job type, and shift type.

The inclusion criteria are as follows:

1. Studies that include participatory organizational interventions.

2. Studies that include participants who were working as of the baseline survey period.

3. Studies that assess mental health or work performance outcomes.

4. Studies that use a cRCT design.

5. Studies published in English or Japanese.

6. Studies published in peer-reviewed journals only (including advanced online publication).

### Information source, search strategy, and data management

Published studies will be searched using the following electronic databases: PubMed, Embase, PsycINFO, PsycArticles, and Japan Medical Abstracts Society. The search terms will include keywords or those associated with some MeSH headings related to the PICO of the studies. The search strategy is shown in Appendix 2. All identified studies will be managed within both EndNote-20 Library and Microsoft Excel files. Before screening the studies, M. Iida will remove duplicate entries by using EndNote-20 Library and export the data to the Excel file.

### Study selection process

We plan to outsource some of the sifting work to specialist contractors. A total of 15 investigators (M. Iida, A. Sakuraya, K.I., H. Asaoka, E.A., A.I., R.I., M. Iwanaga, H.E., Y.O., Y. Kobayashi, Y. Komase, N.S., K.T., and K.W.), and a specialist contractor representative will independently conduct the screening of studies according to the eligibility criteria created earlier in the sifting phase, and the full text of all eligible studies will be obtained. In the full-text review phase, the full texts will be reviewed using a standardized form for assessing eligibility for this study. When resolution cannot be accomplished, the disagreements will be settled by consensus with discussion among all authors. Corresponding authors will be contacted directly if (1) the publication is unclear and may be related to multiple interpretations, or (2) the collected data from the publication do not show data relevant to our study analyses. The reasons for excluding studies will be recorded. A flowchart will be provided to show the entire review process.

### Data collection

Data will be extracted independently from the included studies by 15 investigators (M. Iida, A. Sakuraya, K.I., H. Asaoka, E.A., A.I., R.I., M. Iwanaga, H.E., Y.O., Y. Kobayashi, Y. Komase, N.S., K.T., and K.W.) using a standardized data extraction form. All authors will consult and reach an agreement to resolve any disagreements or inconsistencies. Data of the year of publication, the country in which the study was conducted, the length of follow-up, sample size, demographic characteristics of participants, the contents of intervention, condition of the control group, and outcome variables, and results for mental health or work performance outcomes will be extracted. This extraction form will be piloted and adjusted as needed. Means and standard deviations (SDs) of outcomes at baseline and post-intervention surveys, as well as the number of participants at analyses of intervention and control groups, will be collected for the meta-analysis.

### Risk of bias in individual studies and assessment of metabias

A total of 15 investigators (M. Iida, A. Sakuraya, K.I., H. Asaoka, E.A., A.I., R.I., M. Iwanaga, H.E., Y.O., Y. Kobayashi, Y. Komase, N.S., K.T., and K.W.) will independently assess the study quality of each selected study using the risk-of-bias assessment tool of the Grading of Recommendation Assessment, Development, and Evaluation (GRADE) system.^28^^,^^29^ The risk-of-bias assessment tool of the GRADE system evaluates a cluster-randomized controlled study based on 6 domains: (1a) bias arising from the randomization process; (1b) bias arising from the timing of identification or recruitment of participants; (2) bias due to deviations from intended interventions; (3) bias due to missing outcome data; (4) bias in measurement of the outcome; and (5) bias in selection of the reported result. Each item will be assessed as a low risk of bias, some concerns, or a high risk of bias. Disputes between the 15 evaluators will be resolved by all authors until a consensus is reached. A summary of findings will be created using the GRADE approach to grade the certainty of evidence. Publication bias will be assessed for meta-bias using Egger’s test as well as visually on a funnel plot.

### Data synthesis and statistical methods

#### Primary analyses

For the main analyses, the included studies will be statistically synthesized by a meta-analysis to estimate the pooled effects of the participatory organizational interventions on mental health and work performance outcomes, respectively. The continuous outcomes will be synthesized by calculating SMDs and their 95% confidence intervals (CIs). If the included studies use dichotomous and continuous variables, the continuous outcomes will be converted to dichotomous variables based on appropriate cutoff points and synthesized by calculating odds ratios or relative risks and their 95% confidence intervals (CIs). If there is no reasonable cutoff point, we will analyze dichotomous variables and continuous variables separately. If a meta-analysis cannot be conducted because only 2 or fewer studies are eligible and included, the findings will be presented in narrative form. If no heterogeneity is observed (eg, types of interventions or populations), a fixed-effect model will be used, otherwise a random-effects model will be used. We will assess the heterogeneity by using the Q statistic.^30^

All the collected data and analyzed results will be deposited by the corresponding author and available upon request by external reviewers.

#### Subgroup and sensitivity analyses

Subgroup analyses will be conducted to compare the results under specific contents of intervention (eg, all workers participate in every step of the intervention vs not) and outcome (eg, positive mental health/mental health conditions), if enough data to conduct such analyses can be collected. Any differences between subgroups will be reported, and our findings will be explained in light of these differences. For included studies with a GRADE of low risk, a sensitivity analysis will be conducted.^28^

#### Patient and public involvement

This study has no direct patient or public involvement in its design.

#### Ethics and dissemination

Because this systematic review and meta-analysis is based on previously published studies, it does not require ethical approval. The findings and results will be submitted to and published in a scientific peer-reviewed journal.

## Strengths and limitations

To our knowledge, this will be the first systematic review and meta-analysis to reveal integrated evidence for the effect of participatory organizational intervention on workers’ mental health and work performance. This research will demonstrate how this type of intervention can impact the well-being and productivity of workers. Considering the importance of well-being and productivity at a workplace, the findings of this study will be useful for public and occupational health. Particularly, we will include only cRCT studies in this study. By using cRCTs rather than non-cRCTs we can avoid confounding and selection bias; our systematic review and meta-analysis can examine the effect of participatory organizational intervention with a high level of evidence, which has theoretical significance.

However, this systematic review and meta-analysis study may have some limitations. The generalizability of the findings may be restricted based on the demographic characteristics of the participants included in the selected studies. Furthermore, the selection of databases for this review is based on previous studies, and we are not able to make a comprehensive search. For example, articles in languages other than English or Japanese, and gray literature, such as conference proceedings and unpublished manuscripts, will not be included in this review.

## Supplementary Material

Web_Material_uiae028

## Data Availability

No new data were generated or analyzed in support of this research. The study protocol is registered at the UMIN registry (registration number: UMIN000049453).

## References

[1] World Health Organization . Guidelines on mental health at work. 2022. Accessed May 15, 2023. https://www.who.int/publications/i/item/9789240053052

[2] Forastieri V . Improving Health in the Workplace: ILO’s Framework for Action. Geneva: International Labour Organization (ILO); 2014:4.

[3] International Labour Organization . Occupational Safety and Health Convention (No. 155). 1981.

[4] International Labour Organization . Convention and Recommendation concerning Occupational Health Services. Convention 161 and Recommendation 171. 1985.

[5] Stansfeld SA , North F, White I, Marmot MG. Work characteristics and psychiatric disorder in civil servants in London. J Epidemiol Community Health. 1995;49(1):48–53. 10.1136/jech.49.1.48 7707005 PMC1060074

[6] Stansfeld SA , Fuhrer R, Shipley MJ, Marmot MG. Work characteristics predict psychiatric disorder: prospective results from the Whitehall II study. Occup Environ Med. 1999;56(5):302–307. 10.1136/oem.56.5.302 10472303 PMC1757742

[7] Egan M , Bambra C, Thomas S, Petticrew M, Whitehead M, Thomson H. The psychosocial and health effects of workplace reorganisation. 1. A systematic review of organisational-level interventions that aim to increase employee control. J Epidemiol Community Health. 2007;61(11):945–954. 10.1136/jech.2006.054965 17933951 PMC2465601

[8] Lamontagne AD , Keegel T, Louie AM, Ostry A, Landsbergis PA. A systematic review of the job-stress intervention evaluation literature, 1990–2005. Int J Occup Environ Health. 2007;13(3):268–280. 10.1179/oeh.2007.13.3.268 17915541

[9] Montano D , Hoven H, Siegrist J. Effects of organisational-level interventions at work on employees’ health: a systematic review. BMC Public Health. 2014;14(1):1–9. 10.1186/1471-2458-14-135 24507447 PMC3929163

[10] Nielsen K , Randall R, Holten A-L, González ER. Conducting organizational-level occupational health interventions: what works? Work Stress. 2010;24(3):234–259. 10.1080/02678373.2010.515393

[11] Sakuraya A , Iida M, Imamura K et al. A proposed definition of participatory organizational interventions. J Occup Health. 2023;65(1):e12386. 10.1002/1348-9585.12386 36737041 PMC9897955

[12] Abildgaard JS , Hasson H, von Thiele SU, Løvseth LT, Ala-Laurinaho A, Nielsen K. Forms of participation: the development and application of a conceptual model of participation in work environment interventions. Econ Ind Democr. 2020;41(3):746–769. 10.1177/0143831X17743576

[13] Llorens C , Pérez-Franco J, Oudyk J, et al. COPSOQ III. Guidelines and questionnaire. 2019. https://www.copsoq-network.org/assets/Uploads/COPSOQ-network-guidelines-an-questionnaire-COPSOQ-III-131119-signed.pdf

[14] Tsutsumi A , Shimazu A, Yoshikawa T. Proposed guidelines for primary prevention for mental health at work: an update. Environ Occup Health Pract. 2019;1(1):2–12. 10.1539/eohp.2019-0007-RA

[15] International Organization for Standardization . Occupational health and safety management—psychological health and safety at work. Guidelines for managing psychosocial risks. 2021. Accessed May 15, 2023. https://www.iso.org/obp/ui/#iso:std:iso:45003:ed-1:v1:en

[16] Tsutsumi A , Nagami M, Yoshikawa T, Kogi K, Kawakami N. Participatory intervention for workplace improvements on mental health and job performance among blue-collar workers: a cluster randomized controlled trial. J Occup Environ Med. 2009;51(5):554–563. 10.1097/JOM.0b013e3181a24d28 19365287

[17] Haukka E , Pehkonen I, Leino-Arjas P et al. Effect of a participatory ergonomics intervention on psychosocial factors at work in a randomised controlled trial. Occup Environ Med. 2010;67(3):170–177. 10.1136/oem.2008.043786 19737735

[18] Thibaut J , Walker L. Procedural Justice: A Psychological Analysis. Erlbaum; 1975.

[19] Leventhal GS . What should be done with equity theory? New approaches to the study of fairness in social relationships. In: Gergen KJ, Greenberg MS, Willis RH eds. Social Exchange: Advances in Theory and Research. New York: Plenum Press; 1980:27–55.

[20] Bies RJ , Moag JS. Interactional justice: communication criteria of fairness. In: Lewicki RJ, Sheppard BH, Bazerman MH eds. *Research on Negotiation in Organizations*. Vol 1. JAI Press; 1986:43–55.

[21] Dahl-Jørgensen C , Saksvik PØ. The impact of two organizational interventions on the health of service sector workers. Int J Health Serv. 2005;35(3):529–549. 10.2190/P67F-3U5Y-3DDW-MGT1 16119574

[22] Uchiyama A , Odagiri Y, Ohya Y, Takamiya T, Inoue S, Shimomitsu T. Effect on mental health of a participatory intervention to improve psychosocial work environment: a cluster randomized controlled trial among nurses. J Occup Health. 2013;55(3):173–183. 10.1539/joh.12-0228-OA 23585499

[23] Framke E , Sørensen OH, Pedersen J, Rugulies R. Effect of a participatory organizational-level occupational health intervention on job satisfaction, exhaustion and sleep disturbances: results of a cluster randomized controlled trial. BMC Public Health. 2016;16(1):1210. 10.1186/s12889-016-3871-6 27899101 PMC5129592

[24] Ruotsalainen JH , Verbeek JH, Mariné A, Serra C. Preventing occupational stress in healthcare workers. Cochrane Database Syst Rev. 2014;11:CD002892. 10.1002/14651858.CD002892.pub4 25391582

[25] Reeves BC , Deeks JJ, Higgins JPT, Shea B, Tugwell P, Wells GA. Chapter 24: Including non-randomized studies on intervention effects. In: Higgins JPT, Thomas J, Chandler J, et al., eds. Cochrane Handbook for Systematic Reviews of Interventions version 6.4 (updated August 2023). Cochrane; 2023. Available from www.training.cochrane.org/handbook

[26] Moher D , Shamseer L, Clarke M et al. Preferred reporting items for systematic review and meta-analysis protocols (PRISMA-P) 2015 statement. Syst Rev. 2015;4(1):1–9. 10.1186/2046-4053-4-1 25554246 PMC4320440

[27] Page MJ , McKenzie JE, Bossuyt PM et al. The PRISMA 2020 statement: an updated guideline for reporting systematic reviews. Int J Surg. 2021;88:105906. 10.1016/j.ijsu.2021.105906 33789826

[28] Higgins JPT , Eldridge S, Li T, eds. Chapter 23: Including variants on randomized trials. In Higgins JPT, Thomas J, Chandler J, et al., eds. Cochrane Handbook for Systematic Reviews of Interventions version 6.4. (updated August 2023). Cochrane; 2023. Available from www.training.cochrane.org/handbook.

[29] Sterne JAC , Savović J, Page MJ, et al. RoB 2: a revised tool for assessing risk of bias in randomised trials. BMJ 2019; 366: l4898. 31462531 10.1136/bmj.l4898

[30] Assink M , Wibbelink CJ. Fitting three-level meta-analytic models in R: a step-by-step tutorial. Quant Meth Psych. 2016;12(3):154–174. 10.20982/tqmp.12.3.p154

