# Supplementary material for: Effects of participatory organizational interventions on mental health and work performance: a protocol for systematic review and meta-analysis
Source: J Occup Health. 2024 May 28;66(1):uiae028. doi: 10.1093/joccuh/uiae028 (PMC11272038; doi:10.1093/joccuh/uiae028)
Supplement: Web_Material_uiae028 [file web_material_uiae028.zip › Appendix2_240318.docx]

**Supplementary Appendix 2**

Search terms for PubMed

("Occupational Groups"[Mesh] OR "Occupational Health"[Mesh] OR “enterprise*”[tiab] OR "business*"[tiab] OR "employed"[tiab] OR "employee*"[tiab] OR "employer*"[tiab] OR "employment*"[tiab] OR "informal sector*"[tiab] OR "informal work*"[tiab] OR "laborer*"[tiab] OR "labourer*"[tiab] OR "occupation*"[tiab] OR "personnel*"[tiab] OR "professional*"[tiab] OR "staff*"[tiab] OR "vocation*"[tiab] OR "worker*"[tiab] OR "workforce"[tiab] OR "workplace*"[tiab] OR "work place*"[tiab] OR "worksite*"[tiab] OR "work site*"[tiab] OR "aide*"[tiab] OR "audiologist*"[tiab] OR "ambulance*"[tiab] OR "care assistant*"[tiab] OR "clinician*"[tiab] OR "coastguard*"[tiab] OR "coast guard*"[tiab] OR "dentist*"[tiab] OR "detective*"[tiab] OR "disaster responder*"[tiab] OR "doctor*"[tiab] OR "domestic health car*"[tiab] OR "domiciliary car*"[tiab] OR "emergency service*"[tiab] OR "emergency responder*"[tiab] OR "emergency medical"[tiab] OR "firefighter*"[tiab] OR "fire fighter*"[tiab] OR "first responder*"[tiab] OR "general practitioner*"[tiab] OR "health care provider*"[tiab] OR "healthcare provider*"[tiab] OR "health visitor*"[tiab] OR "home care service*"[tiab] OR "humanitarian aid*"[tiab] OR "humanitarian relie*"[tiab] OR "humanitarian service*"[tiab] OR "law enforc*"[tiab] OR "lifeguard*"[tiab] OR "life guard*"[tiab] OR "medical resident*"[tiab] OR "medic"[tiab] OR "medics"[tiab] OR "nurse*"[tiab] OR "nursing"[tiab] OR "midwi*"[tiab] OR "paramedic*"[tiab] OR "policemen"[tiab] OR "policeman"[tiab] OR "police men"[tiab] OR "police man"[tiab] OR "police women"[tiab] OR "police woman"[tiab] OR "police officer*"[tiab] OR "firemen"[tiab] OR "fireman"[tiab] OR "fire men"[tiab] OR "fire man"[tiab] OR "fire women"[tiab] OR "fire woman"[tiab] OR "pharmacis*"[tiab] OR "psychologist*"[tiab] OR "physician*"[tiab] OR "practitioner*"[tiab] OR "relief work*"[tiab] OR "rescuer*"[tiab] OR "rescue work*"[tiab] OR "therapist*"[tiab] OR "veterinaria*"[tiab])

AND

("Employee Grievances"[Mesh] OR "Work Schedule Tolerance"[Mesh]OR "Bullying"[Mesh] OR "Interpersonal Relations"[Mesh] OR "Prejudice"[Mesh] OR "Social Discrimination"[Mesh] OR "harassment, non-sexual"[Mesh] OR “risk management”[Mesh] OR "Organizational Culture"[Mesh] OR “Organizational Policy”[MeSH] OR “leadership”[Mesh] OR "Personnel Downsizing"[Mesh] OR "Staff Development"[Mesh] OR "Employee Performance Appraisal"[Mesh] OR “inservice training*”[tiab] OR “in service training*”[tiab] OR "organizational intervention*"[tiab] OR "organisational intervention*"[tiab] OR “participatory intervention*”[tiab] OR “team intervention*”[tiab] OR “compressed hour*”[tiab] OR "compressed work*"[tiab] OR “compressed week*”[tiab] OR “day-time”[Tiab] OR “daytime”[tiab] OR “day time”[tiab] OR “flexible schedule*”[tiab] OR “flexible work*”[tiab] OR “inflexible schedule*”[tiab] OR “inflexible work*”[tiab] OR “full-time”[Tiab] OR “gig economy”[tiab] OR “long hour*”[tiab] OR “night-time”[Tiab] OR “nighttime”[tiab] OR “night time”[tiab] OR “night shift*”[tiab] OR “overtime*”[tiab] OR “part-time”[Tiab] OR “recovery”[tiab] OR “remote work*”[tiab] OR “shift work*”[Tiab] OR “self-scheduling”[tiab] OR “temporary work*”[Tiab] OR “work schedule*”[tiab] OR “working schedule*”[tiab] OR “working hour*”[Tiab] OR “work hour*” [tiab] OR “working time”[Tiab] OR “work shift*”[Tiab] OR “zero hour*”[tiab] OR ("life"[tiab] AND “balance"[tiab]) OR (“work*”[tiab] AND "life"[tiab] AND “balance"[tiab]) OR ("life"[tiab] AND “family"[tiab]) OR ("reconciling"[tiab] AND “work*"[tiab]) OR ("living"[tiab] AND "working"[tiab]) OR “work overload*”[Tiab] OR “work over-load*”[Tiab] OR “work pace”[tiab] OR “time pressure*”[Tiab] OR “decision latitude”[Tiab] OR “demand resource*”[Tiab ] OR “effort reward*”[Tiab] OR “high demand*”[Tiab] OR “job control”[Tiab] OR “job demand*”[Tiab] OR “job strain”[Tiab] OR “lack of control”[Tiab] OR “task restructur*”[tiab] OR “low control”[Tiab] OR “work demand*”[Tiab] OR “work control”[Tiab] OR “work influence*”[Tiab] OR “work strain”[Tiab] OR “boredom”[Tiab] OR “coping”[Tiab] OR ((“control”[tiab] OR “unpleasant”[tiab] OR “aversive”[tiab]) AND “task*”[tiab]) OR “job content”[tiab] OR monoton*[tiab] OR “under stimulat*”[tiab] OR “ageism”[Tiab] OR “aggression”[tiab] OR “bullying”[Tiab] OR “discrimination”[Tiab] OR “interpersonal relation*”[Tiab] OR “harass*”[Tiab] OR “homophobia”[Tiab] OR “microaggression”[tiab] OR “prejudice”[tiab] OR “racism”[Tiab] OR “sexism”[Tiab] OR “silent workplace*”[Tiab] OR “social capital” [Tiab] OR “solitary work*” [tiab] OR “isolated work*” [tiab] OR “supervision”[tiab] “victimization*”[Tiab] OR “work place conflict*”[Tiab] OR “workplace violen*”[Tiab] OR “work place violen*”[Tiab] OR "lean management"[tiab] OR "risk management"[tiab] OR “safety management”[tiab] OR “work environment”[tiab] OR “working environment”[tiab] OR “work condition*”[tiab] OR “working condition*”[tiab] OR “work organization”[tiab] OR “work design”[tiab] OR “communication*”[tiab] OR “organisational culture”[tiab] OR “organizational culture”[tiab] OR “organisational function”[tiab] OR “organizational function”[tiab] OR “organisational injustice*”[Tiab] OR “organizational injustice*”[Tiab] OR “health and safety”[tiab] OR “organisational justice*”[Tiab] OR “organizational justice*”[Tiab] OR “leadership”[tiab] OR “lean production”[Tiab] OR "lean management"[tiab] OR “labour relation*”[tiab] OR “management practice*”[tiab] OR “management culture”[tiab] OR “management measure*”[tiab] OR ((“participation”[tiab] OR “involvement”[tiab]) AND “decision making*”[tiab]) OR “organisational policy”[tiab] OR “organizational policy”[tiab] OR “organisational policies”[tiab] OR “organizational policies”[tiab] OR “psychosocial risk*”[tiab] OR “procedural justice”[tiab] OR “procedural injustice”[tiab] OR “clear role”[tiab] OR “skill discretion*”[Tiab] OR “role ambiguity”[Tiab] OR “role conflict*”[Tiab] OR “role clarity”[tiab] OR “unclear role”[tiab] OR “work role*”[Tiab] OR “career development”[tiab] OR “job security”[Tiab] OR “job insecurity”[Tiab] OR “over skilled”[tiab] OR “performance evaluation”[tiab] OR “professional development”[tiab] OR ((“work”[tiab] OR “job”[tiab]) AND “promotion”[tiab]) OR “staff development”[Tiab] OR “under skilled”[tiab] OR “team building”[tiab] OR “teambuilding”[tiab] OR (“team*”[tiab] AND “participatory”[tiab]) OR “teamwork*”[tiab] OR ((“team*”[tiab] OR “co worker”[tiab] OR “colleague*”[tiab] ) AND (“work”[tiab] OR “building”[tiab] OR “program*”[tiab])))

AND

(randomized controlled trial [pt] OR (randomized [tiab] AND controlled [tiab] AND trial [tiab]))

AND

(("Mental Disorders"[Mesh] OR "Mental Health"[Mesh] OR "Psychology, Industrial"[Mesh] OR "Stress, Psychological"[Mesh] OR "adjustment"[tiab] OR "affective disorder*"[tiab] OR "anxiet*"[tiab] OR "bipolar*"[tiab] OR "burn out*"[tiab] OR "burnout*"[tiab] OR "CMD" [tiab] OR "depressi*"[tiab] OR "eating disorder*"[tiab] OR "mental disorder*"[tiab] OR "mental health*"[tiab] OR "mental illness*"[tiab] OR "mood disorder*"[tiab] OR "obsessive compulsive disorder*"[tiab] OR "ocd"[tiab] OR "panic disorder*"[tiab] OR "phobi*"[tiab] OR "post traumatic*"[tiab] OR "psychiatric diagnos*"[tiab] OR "psychiatric disease*"[tiab] OR "psychiatric disorder*"[tiab] OR "psychiatric illness*"[tiab] OR "psychological disorder*"[tiab] OR "psychos*"[tiab] OR "psychotic*"[tiab] OR "psychological distress*"[tiab] OR "ptsd"[tiab] OR "ptss"[tiab] OR "somatoform disorder*"[tiab] OR "schizophren*"[tiab] OR "stress*"[tiab]) OR

("Optimism"[Mesh] OR "Personal Satisfaction"[Mesh] OR "Self Concept"[Mesh:NoExp] OR "Self Efficacy"[Mesh] OR "Self-Control"[Mesh] OR "life engag*"[tiab] OR "life satisf*"[tiab] OR "meaning of life"[tiab] OR "purpose in life"[tiab] OR "positive affect*"[tiab] OR "positive emotion*"[tiab] OR "resilien*"[tiab] OR "self concept*"[tiab] OR "self control*"[tiab] OR "self efficac*"[tiab] OR "self esteem*"[tiab] OR "swb"[tiab] OR "well being*"[tiab] OR "wellbeing*"[tiab] 　OR "Job Satisfaction"[MeSH] OR "job satisf*"[tiab] OR "work satisf*"[tiab] OR "work engag*"[tiab]) OR

("Work Capacity Evaluation"[Mesh] OR "effectiveness"[tiab] OR "employabil*"[tiab] OR "presenteeism*"[tiab] OR "productivit*"[tiab] OR "work abilit*"[tiab] OR "work capacit*"[tiab] OR "work disabilit*"[tiab] OR "work function*"[tiab] OR "work participati*"[tiab] OR "work performan*"[tiab]))

Search terms for PsycINFO

(MA "Occupational Groups" OR MA "Occupational Health" OR TI “enterprise*” OR TI "business*" OR TI "employed" OR TI "employee*" OR TI "employer*" OR TI "employment*" OR TI "informal sector*" OR TI "informal work*" OR TI "laborer*" OR TI "labourer*" OR TI "occupation*" OR TI "personnel*" OR TI "professional*" OR TI "staff*" OR TI “vocation*” OR TI "worker*" OR TI "workforce" OR TI "workplace*" OR TI "work place*" OR TI "worksite*" OR TI "work site*" OR TI "aide*" OR TI "audiologist*" OR TI "ambulance*" OR TI "care assistant*" OR TI "clinician*" OR TI "coastguard*" OR TI "coast guard*" OR TI "dentist*" OR TI "detective*" OR TI "disaster responder*" OR TI "doctor*" OR TI "domestic health car*" OR TI "domiciliary car*" OR TI "emergency service*" OR TI "emergency responder*" OR TI "emergency medical" OR TI "firefighter*" OR TI "fire fighter*" OR TI "first responder*" OR TI general practitioner*" OR TI "health care provider*" OR TI “healthcare provider*” OR TI "health visitor*" OR TI "home care service*" OR TI "humanitarian aid*" OR TI “humanitarian relie*” OR TI "humanitarian service*" OR TI "law enforc*" OR TI "lifeguard*" OR TI "life guard*" OR TI "medical resident*" OR TI "medic" OR TI "medics" OR TI "nurse*" OR TI "nursing" OR TI "midwi*" OR TI "paramedic*" OR "TI policemen" OR TI "policeman" OR "TI police men” OR TI "police man" OR TI "police women" OR TI "police woman" OR TI "police officer*" OR TI "firemen" OR "TI fireman” OR TI "fire men" OR TI "fire man" OR TI "fire women" OR TI "fire woman" OR TI “pharmacis*” OR TI "psychologist*" OR TI "physician*" OR TI "practitioner*" OR TI “relief work*” OR TI "rescuer*" OR TI "rescue work*" OR TI “therapist*” OR RI “veterinaria*” OR AB “enterprise*” OR AB "business*" OR AB"employed" OR AB "employee*" OR AB "employer*" OR AB "employment*" OR AB "informal sector*" OR AB "informal work*" OR AB "laborer*" OR AB "labourer*" OR AB "occupation*" OR AB "personnel*" OR AB "professional*" OR AB "staff*" OR AB “vocation*” OR AB "worker*" OR AB "workforce" OR AB "workplace*" OR AB "work place*" OR AB "worksite*" OR AB "work site*" OR AB "aide*" OR AB "audiologist*" OR AB "ambulance*" OR AB "care assistant*" OR AB "clinician*" OR AB "coastguard*" OR AB "coast guard*" OR AB "dentist*" OR AB "detective*" OR AB "disaster responder*" OR AB "doctor*" OR AB "domestic health car*" OR AB "domiciliary car*" OR AB "emergency service*" OR AB "emergency responder*" OR AB "emergency medical" OR AB "firefighter*" OR AB "fire fighter*" OR AB "first responder*" OR AB general practitioner*" OR AB "health care provider*" OR AB “healthcare provider*” OR AB "health visitor*" OR AB "home care service*" OR AB "humanitarian aid*" OR AB “humanitarian relie*” OR AB "humanitarian service*" OR AB "law enforc*" OR AB "lifeguard*" OR AB "life guard*" OR AB "medical resident*" OR AB "medic" OR AB "medics" OR AB "nurse*" OR AB "nursing" OR AB "midwi*" OR AB "paramedic*" OR "AB policemen" OR AB "policeman" OR "AB police men” OR AB "police man" OR AB "police women" OR AB "police woman" OR AB "police officer*" OR AB "firemen" OR "AB fireman” OR AB "fire men" OR AB "fire man" OR AB "fire women" OR AB "fire woman" OR AB “pharmacis*” OR AB "psychologist*" OR AB "physician*" OR AB "practitioner*" OR AB “relief work*” OR AB "rescuer*" OR AB "rescue work*" OR AB “therapist*” OR AB "veterinaria*")

AND

(MA "Employee Grievances" OR MA "Work Schedule Tolerance" OR MA "Bullying" OR MA "Interpersonal Relations" OR MA "Prejudice" OR MA "Social Discrimination" OR MA "harassment, non-sexual" OR MA “risk management” OR MA "Organizational Culture" OR MA “Organizational Policy” OR MA “leadership” OR MA "Personnel Downsizing" OR MA "Staff Development" OR MA "Employee Performance Appraisal" OR TI “inservice training*” OR TI “in service training*” OR TI "organizational intervention*" OR TI "organisational intervention*" OR TI “participatory intervention*” OR TI “team intervention*” OR AB “inservice training*” OR AB “in service training*” OR AB "organizational intervention*" OR AB "organisational intervention*" OR AB “participatory intervention*” OR AB “team intervention*” OR TI “compressed hour*” OR TI "compressed work*" OR TI “compressed week*” OR TI “day-time” OR TI“daytime” OR TI “day time” OR TI “flexible schedule*” OR TI “flexible work*” OR TI “inflexible schedule*” OR TI “inflexible work*” OR TI “full-time” OR TI “gig economy” OR TI “long hour*” OR TI “night-time” OR TI “nighttime” OR TI “night time” OR TI “night shift*” OR TI “overtime*” OR TI “part-time” OR TI recovery OR TI “remote work*” OR TI “shift work*” OR TI “self-scheduling” OR TI “temporary work*” OR TI “work schedule*” OR TI “working schedule*” OR TI “working hour*” OR TI “work hour*” OR TI “working time” OR TI “work shift*” OR TI “zero hour*” OR (TI "life" AND TI “balance") OR (TI “work*” AND TI "life" AND TI “balance") OR (TI "life" AND TI “family") OR (TI "reconciling" AND TI “work*") OR (TI "living" AND TI "working") OR TI “work overload*” OR TI “work over-load*” OR TI “work pace” OR TI “time pressure*” OR TI “decision latitude” OR TI “demand resource*” OR TI “effort reward*” OR TI “high demand*” OR TI “job control” OR TI “job demand*” OR TI “job strain” OR TI “lack of control” OR TI “task restructur*” OR TI “low control” OR TI “work demand*” OR TI “work control” OR TI “work influence*” OR TI “work strain” OR TI “boredom” OR TI “coping” OR ((TI “control” OR TI “unpleasant” OR TI “aversive”) AND TI “task*”) OR TI “job content” OR TI monoton* OR TI “under stimulat*” OR AB “compressed hour*” OR AB "compressed work*" OR AB “compressed week*” OR AB “day-time” OR AB “daytime” OR AB “day time” OR AB “flexible schedule*” OR AB “flexible work*” OR AB “inflexible schedule*” OR AB “inflexible work*” OR AB “full-time” OR AB “gig economy” OR AB “long hour*” OR AB “night-time” OR AB “nighttime” OR AB “night time” OR AB “night shift*” OR AB “overtime*” OR AB “part-time” OR AB recovery OR AB “remote work*” OR AB “shift work*” OR AB “self-scheduling” OR AB “temporary work*” OR AB “work schedule*” OR AB “working schedule*” OR AB “working hour*” OR AB “work hour*” OR AB “working time” OR AB “work shift*” OR AB “zero hour*” OR (AB "life" AND AB “balance") OR (AB “work*” AND AB "life" AND AB “balance") OR (AB "life" AND AB “family") OR (AB "reconciling" AND AB “work*") OR (AB "living" AND AB "working") OR AB “work overload*” OR AB “work over-load*” OR AB “work pace” OR AB “time pressure*” OR AB “decision latitude” OR AB “demand resource*” OR AB “effort reward*” OR AB “high demand*” OR AB “job control” OR AB “job demand*” OR AB “job strain” OR AB “lack of control” OR AB “task restructur*” OR AB “low control” OR AB “work demand*” OR AB “work control” OR AB “work influence*” OR AB “work strain” OR AB “boredom” OR AB “coping” OR ((AB “control” OR AB “unpleasant” OR AB “aversive”) AND AB “task*”) OR AB “job content” OR AB monoton* OR AB “under stimulat*” OR TI “ageism” OR TI “aggression” OR TI “bullying” OR TI “discrimination” OR TI “interpersonal relation*” OR TI “harass*” OR TI “homophobia” OR TI “microaggression” OR TI “prejudice” OR TI “racism” OR TI “sexism” OR TI “silent workplace*” OR TI “social capital” OR TI “solitary work*” OR TI “isolated work*” OR TI “supervision” TI “victimization*” OR TI “work place conflict*” OR TI “workplace violen*” OR TI “work place violen*” OR TI "lean management" OR AB “ageism” OR AB “aggression” OR AB “bullying” OR AB “discrimination” OR AB “interpersonal relation*” OR AB “harass*” OR AB “homophobia” OR AB “microaggression” OR AB “prejudice” OR AB “racism” OR AB “sexism” OR AB “silent workplace*” OR AB “social capital” OR AB “solitary work*” OR AB “isolated work*” OR AB “supervision” AB “victimization*” OR AB “work place conflict*” OR AB “workplace violen*” OR AB “work place violen*” OR AB "lean management" OR TI "risk management" OR TI “safety management” OR TI “work environment” OR TI “working environment” OR TI “work condition*” OR TI “working condition*” OR TI “work organization” OR TI “work design” OR AB "risk management" OR AB “safety management” OR AB “work environment” OR AB “working environment” OR AB “work condition*” OR AB “working condition*” OR AB “work organization” OR AB “work design” OR TI “communication*” OR TI “organisational culture” OR TI “organizational culture” OR TI “organisational function” OR TI “organizational function” OR TI “organisational injustice*” OR TI “organizational injustice*” OR TI “health and safety” OR TI “organisational justice*” OR TI “organizational justice*” OR TI “leadership” OR TI “lean production” OR TI "lean management" OR TI “labour relation*” OR TI “management practice*” OR TI “management culture” OR TI “management measure*” OR ((TI “participation” OR TI “involvement”) AND TI “decision making*”) OR TI “organisational policy” OR TI “organizational policy” OR TI “organisational policies” OR TI “organizational policies” OR TI “psychosocial risk*” OR TI “procedural justice” OR TI “procedural injustice” OR TI “clear role” OR TI “skill discretion*” OR TI “role ambiguity” OR TI “role conflict*” OR TI “role clarity” OR TI “unclear role” OR TI “work role*” OR AB “communication*” OR AB “organisational culture” OR AB “organizational culture” OR AB “organisational function” OR AB “organizational function” OR AB “organisational injustice*” OR AB “organizational injustice*” OR AB “health and safety” OR AB “organisational justice*” OR AB “organizational justice*” OR AB “leadership” OR AB “lean production” OR AB "lean management" OR AB “labour relation*” OR AB “management practice*” OR AB “management culture” OR AB “management measure*” OR ((AB “participation” OR AB “involvement”) AND AB “decision making*”) OR AB “organisational policy” OR AB “organizational policy” OR AB “organisational policies” OR AB “organizational policies” OR AB “psychosocial risk*” OR AB “procedural justice” OR AB “procedural injustice” OR AB “clear role” OR AB “skill discretion*” OR AB “role ambiguity” OR AB “role conflict*” OR AB “role clarity” OR AB “unclear role” OR AB “work role*” OR TI “career development” OR TI “job security” OR TI “job insecurity” OR TI “over skilled” OR TI “performance evaluation” OR TI “professional development” OR ((TI “work” OR TI “job”) AND TI “promotion”) OR TI “staff development” OR TI “under skilled” OR TI “team building” OR TI “teambuilding” OR ( TI “team*” AND TI “participatory”) OR TI “teamwork*” OR (( TI “team*” OR TI “co worker” OR TI “colleague*” ) AND (TI “work” OR TI “building” OR TI “program*”)) OR AB “career development” OR AB “job security” OR AB “job insecurity” OR AB “over skilled” OR AB “performance evaluation” OR AB “professional development” OR ((AB “work” OR AB “job”) AND AB “promotion”) OR AB “staff development” OR AB “under skilled” OR AB “team building” OR AB “teambuilding” OR ( AB “team*” AND AB “participatory”) OR AB “teamwork*” OR (( AB “team*” OR AB “co worker” OR AB “colleague*” ) AND (AB “work” OR AB “building” OR AB “program*”)))

AND

((TI randomized AND TI controlled AND TI trial) OR (AB randomized AND AB controlled AND AB trial))

AND

((MA "Mental Disorders" OR MA "Mental Health" OR MA "Psychology, Industrial" OR MA "Stress, Psychological" OR TI "adjustment" OR TI "affective disorder*" OR TI "anxiet*" OR TI "bipolar*" OR TI "burn out*" OR TI "burnout*" OR TI "CMD" OR TI "depressi*" OR TI "eating disorder*" OR TI "mental disorder*" OR TI "mental health*" OR TI "mental illness*" OR TI "mood disorder*" OR TI "obsessive compulsive disorder*" OR TI "ocd" OR TI "panic disorder*" OR TI "phobi*" OR TI "post traumatic*" OR TI "psychiatric diagnos*" OR TI "psychiatric disease*" OR TI "psychiatric disorder*" OR TI "psychiatric illness*" OR TI "psychological disorder*" OR TI "psychos*" OR TI "psychotic*" OR TI "psychological distress*" OR TI "ptsd" OR TI "ptss" OR TI "somatoform disorder*" OR TI "schizophren*" OR TI "stress*" OR AB "adjustment" OR AB "affective disorder*" OR AB "anxiet*" OR AB "bipolar*" OR AB "burn out*" OR AB "burnout*" OR AB "CMD" OR AB "depressi*" OR AB "eating disorder*" OR AB "mental disorder*" OR AB "mental health*" OR AB "mental illness*" OR AB "mood disorder*" OR AB "obsessive compulsive disorder*" OR AB "ocd" OR AB "panic disorder*" OR AB "phobi*" OR AB "post traumatic*" OR AB "psychiatric diagnos*" OR AB "psychiatric disease*" OR AB "psychiatric disorder*" OR AB "psychiatric illness*" OR AB "psychological disorder*" OR AB "psychos*" OR AB "psychotic*" OR AB "psychological distress*" OR AB "ptsd" OR AB "ptss" OR AB "somatoform disorder*" OR AB "schizophren*" OR AB "stress*" ) OR

(MA "Optimism" OR MA "Personal Satisfaction" OR MA "Self Concept" OR MA "Self Efficacy" OR MA "Self-Control" OR TI "life engag*" OR TI "life satisf*" OR TI "meaning of life" OR TI "purpose in life" OR TI "positive affect*" OR TI "positive emotion*" OR TI "resilien*" OR TI "self concept*" OR TI "self control*" OR TI "self efficac*" OR TI "self esteem*" OR TI "swb" OR TI "well being*" OR TI "wellbeing*" OR AB "life engag*" OR AB "life satisf*" OR AB "meaning of life" OR AB "purpose in life" OR AB "positive affect*" OR AB "positive emotion*" OR AB "resilien*" OR AB "self concept*" OR AB "self control*" OR AB "self efficac*" OR AB "self esteem*" OR AB "swb" OR AB "well being*" OR AB "wellbeing*" OR MA "Job Satisfaction" OR TI "job satisf*" OR TI "work satisf*" OR TI "work engag*" AB "job satisf*" OR AB "work satisf*" OR AB "work engag*") OR

(MA "Work Capacity Evaluation" OR TI "effectiveness" OR TI "employabil*" OR TI "presenteeism*" OR TI "productivit*" OR TI "work abilit*" OR TI "work capacit*" OR TI "work disabilit*" OR TI "work function*"OR TI "work participati*" OR TI "work performan*" OR AB "effectiveness" OR AB "employabil*" OR AB "presenteeism*" OR AB "productivit*" OR AB "work abilit*" OR AB "work capacit*" OR AB "work disabilit*" OR AB "work function*"OR AB "work participati*" OR AB "work performan*" ))

Search terms for EMBASE

(‘occupational groups’/de OR ‘occupational health’/de OR ‘enterprise*’:ti,ab OR ‘business*’:ti,ab OR ‘employed’:ti,ab OR ‘employee*’:ti,ab OR ‘employer*’:ti,ab OR ‘employment*’:ti,ab OR ‘informal sector*’:ti,ab OR ‘informal work*’:ti,ab OR ‘laborer*’:ti,ab OR ‘labourer*’:ti,ab OR ‘occupation*’:ti,ab OR ‘personnel*’:ti,ab OR ‘professional*’:ti,ab OR ‘staff*’:ti,ab OR ‘vocation*’:ti,ab OR ‘worker*’:ti,ab OR ‘workforce’:ti,ab OR ‘workplace*’:ti,ab OR ‘work place*’:ti,ab OR ‘worksite*’:ti,ab OR ‘work site*’:ti,ab OR ‘aide*’:ti,ab OR ‘audiologist*’:ti,ab OR ‘ambulance*’:ti,ab OR ‘care assistant*’:ti,ab OR ‘clinician*’:ti,ab OR ‘coastguard*’:ti,ab OR ‘coast guard*’:ti,ab OR ‘dentist*’:ti,ab OR ‘detective*’:ti,ab OR ‘disaster responder*’:ti,ab OR ‘doctor*’:ti,ab OR ‘domestic health car*’:ti,ab OR ‘domiciliary car*’:ti,ab OR ‘emergency service*’:ti,ab OR ‘emergency responder*’:ti,ab OR ‘emergency medical’:ti,ab OR ‘firefighter*’:ti,ab OR ‘fire fighter*’:ti,ab OR ‘first responder*’:ti,ab OR ‘general practitioner*’:ti,ab OR ‘health care provider*’:ti,ab OR ‘healthcare provider*’:ti,ab OR ‘health visitor*’:ti,ab OR ‘home care service*’:ti,ab OR ‘humanitarian aid*’:ti,ab OR ‘humanitarian relie*’:ti,ab OR ‘humanitarian service*’:ti,ab OR ‘law enforc*’:ti,ab OR ‘lifeguard*’:ti,ab OR ‘life guard*’:ti,ab OR ‘medical resident*’:ti,ab OR ‘medic’:ti,ab OR ‘medics’:ti,ab OR ‘nurse*’:ti,ab OR ‘nursing’:ti,ab OR ‘midwi*’:ti,ab OR ‘paramedic*’:ti,ab OR ‘policemen’:ti,ab OR ‘policeman’:ti,ab OR ‘police men’:ti,ab OR ‘police man’:ti,ab OR ‘police women’:ti,ab OR ‘police woman’:ti,ab OR ‘police officer*’:ti,ab OR ‘firemen’:ti,ab OR ‘fireman’:ti,ab OR ‘fire men’:ti,ab OR ‘fire man’:ti,ab OR ‘fire women’:ti,ab OR ‘fire woman’:ti,ab OR ‘pharmacis*’:ti,ab OR ‘psychologist*’:ti,ab OR ‘physician*’:ti,ab OR ‘practitioner*’:ti,ab OR ‘relief work*’:ti,ab OR ‘rescuer*’:ti,ab OR ‘rescue work*’:ti,ab OR ‘therapist*’:ti,ab OR ‘veterinaria*’:ti,ab)

AND

(‘employee grievances’/de OR ‘work schedule tolerance’:de OR ‘bullying’/de OR ‘interpersonal relations’/de OR ‘prejudice’/de OR ‘social discrimination’/de OR ‘harassment, non-sexual’/de OR ‘risk management’/de OR ‘organizational culture’/de OR ‘organizational policy’/de OR ‘leadership’/de OR ‘personnel downsizing’/de OR ‘staff development’/de OR ‘employee performance appraisal’/de OR ‘inservice training*’:ti,ab OR ‘in service training*’:ti,ab OR ‘organizational intervention*’:ti,ab OR ‘organisational intervention*’:ti,ab OR ‘participatory intervention*’:ti,ab OR ‘team intervention*’:ti,ab OR ‘compressed hour*’:ti,ab OR ‘compressed work*’:ti,ab OR ‘compressed week*’:ti,ab OR ‘day-time’:ti,ab OR ‘daytime’:ti,ab OR ‘day time’:ti,ab OR ‘flexible schedule*’:ti,ab OR ‘flexible work*’:ti,ab OR ‘inflexible schedule*’:ti,ab OR ‘inflexible work*’:ti,ab OR ‘full-time’:ti,ab OR ‘gig economy’:ti,ab OR ‘long hour*’:ti,ab OR ‘night-time’:ti,ab OR ‘nighttime’:ti,ab OR ‘night time’:ti,ab OR ‘night shift*’:ti,ab OR ‘overtime*’:ti,ab OR ‘part-time’:ti,ab OR ‘recovery’:ti,ab OR ‘remote work*’:ti,ab OR ‘shift work*’:ti,ab OR ‘self-scheduling’:ti,ab OR ‘temporary work*’:ti,ab OR ‘work schedule*’:ti,ab OR ‘working schedule*’:ti,ab OR ‘working hour*’:ti,ab OR ‘work hour*’:ti,ab OR ‘working time’:ti,ab OR ‘work shift*’:ti,ab OR ‘zero hour*’:ti,ab OR (‘life’:ti,ab AND ‘balance’:ti,ab) OR (‘work*’:ti,ab AND ‘life’:ti,ab AND ‘balance’:ti,ab) OR (‘life’:ti,ab AND ‘family’:ti,ab) OR (‘reconciling’:ti,ab AND ‘work*’:ti,ab) OR (‘living’:ti,ab AND ‘working’:ti,ab) OR ‘work overload*’:ti,ab OR ‘work over-load*’:ti,ab OR ‘work pace’:ti,ab OR ‘time pressure*’:ti,ab OR ‘decision latitude’:ti,ab OR ‘demand resource*’:ti,ab OR ‘effort reward*’:ti,ab OR ‘high demand*’:ti,ab OR ‘job control’:ti,ab OR ‘job demand*’:ti,ab OR ‘job strain’:ti,ab OR ‘lack of control’:ti,ab OR ‘task restructur*’:ti,ab OR ‘low control’:ti,ab OR ‘work demand*’:ti,ab OR ‘work control’:ti,ab OR ‘work influence*’:ti,ab OR ‘work strain’:ti,ab OR ‘boredom’:ti,ab OR ‘coping’:ti,ab OR ((‘control’:ti,ab OR ‘unpleasant’:ti,ab OR ‘aversive’:ti,ab) AND ‘task*’:ti,ab) OR ‘job content’:ti,ab OR monoton*:ti,ab OR ‘under stimulat*’:ti,ab OR ‘ageism’:ti,ab OR ‘aggression’:ti,ab OR ‘bullying’:ti,ab OR ‘discrimination’:ti,ab OR ‘interpersonal relation*’:ti,ab OR ‘harass*’:ti,ab OR ‘homophobia’:ti,ab OR ‘microaggression’:ti,ab OR ‘prejudice’:ti,ab OR ‘racism’:ti,ab OR ‘sexism’:ti,ab OR ‘silent workplace*’:ti,ab OR ‘social capital’:ti,ab OR ‘solitary work*’:ti,ab OR ‘isolated work*’:ti,ab OR ‘supervision’:ti,ab ‘victimization*’:ti,ab OR ‘work place conflict*’:ti,ab OR ‘workplace violen*’:ti,ab OR ‘work place violen*’:ti,ab OR ‘lean management’:ti,ab OR ‘risk management’:ti,ab OR ‘safety management’:ti,ab OR ‘work environment’:ti,ab OR ‘working environment’:ti,ab OR ‘work condition*’:ti,ab OR ‘working condition*’:ti,ab OR ‘work organization’:ti,ab OR ‘work design’:ti,ab OR ‘communication*’:ti,ab OR ‘organisational culture’:ti,ab OR ‘organizational culture’:ti,ab OR ‘organisational function’:ti,ab OR ‘organizational function’:ti,ab OR ‘organisational injustice*’:ti,ab OR ‘organizational injustice*’:ti,ab OR ‘health and safety’:ti,ab OR ‘organisational justice*’:ti,ab OR ‘organizational justice*’:ti,ab OR ‘leadership’:ti,ab OR ‘lean production’:ti,ab OR ‘lean management’:ti,ab OR ‘labour relation*’:ti,ab OR ‘management practice*’:ti,ab OR ‘management culture’:ti,ab OR ‘management measure*’:ti,ab OR ((‘participation’:ti,ab OR ‘involvement’:ti,ab) AND ‘decision making*’:ti,ab) OR ‘organisational polic*’:ti,ab OR ‘organizational polic*’:ti,ab OR ‘psychosocial risk*’:ti,ab OR ‘procedural justice’:ti,ab OR ‘procedural injustice’:ti,ab OR ‘clear role’:ti,ab OR ‘skill discretion*’:ti,ab OR ‘role ambiguity’:ti,ab OR ‘role conflict*’:ti,ab OR ‘role clarity’:ti,ab OR ‘unclear role’:ti,ab OR ‘work role*’:ti,ab OR ‘career development’:ti,ab OR ‘job security’:ti,ab OR ‘job insecurity’:ti,ab OR ‘over skilled’:ti,ab OR ‘performance evaluation’:ti,ab OR ‘professional development’:ti,ab OR ((‘work’:ti,ab OR ‘job’:ti,ab) AND ‘promotion’:ti,ab) OR ‘staff development’:ti,ab OR ‘under skilled’:ti,ab OR ‘team building’:ti,ab OR ‘teambuilding’:ti,ab OR (‘team*’:ti,ab AND ‘participatory’:ti,ab) OR ‘teamwork*’:ti,ab OR ((‘team*’:ti,ab OR ‘co worker’:ti,ab OR ‘colleague*’:ti,ab ) AND (‘work’:ti,ab OR ‘building’:ti,ab OR ‘program*’:ti,ab)))

AND

(randomized:ti,ab AND controlled:ti,ab AND trial:ti,ab)

AND

((‘mental disorders’/de OR ‘mental health’/de OR ‘psychology, industrial’/de OR ‘stress, psychological’/de OR ‘adjustment’:ti,ab OR ‘affective disorder*’:ti,ab OR ‘anxiet*’:ti,ab OR ‘bipolar*’:ti,ab OR ‘burn out*’:ti,ab OR ‘burnout*’:ti,ab OR ‘cmd’:ti,ab OR ‘depressi*’:ti,ab OR ‘eating disorder*’:ti,ab OR ‘mental disorder*’:ti,ab OR ‘mental health*’:ti,ab OR ‘mental illness*’:ti,ab OR ‘mood disorder*’:ti,ab OR ‘obsessive compulsive disorder*’:ti,ab OR ‘ocd’:ti,ab OR ‘panic disorder*’:ti,ab OR ‘phobi*’:ti,ab OR ‘post traumatic*’:ti,ab OR ‘psychiatric diagnos*’:ti,ab OR ‘psychiatric disease*’:ti,ab OR ‘psychiatric disorder*’:ti,ab OR ‘psychiatric illness*’:ti,ab OR ‘psychological disorder*’:ti,ab OR ‘psychos*’:ti,ab OR ‘psychotic*’:ti,ab OR ‘psychological distress*’:ti,ab OR ‘ptsd’:ti,ab OR ‘ptss’:ti,ab OR ‘somatoform disorder*’:ti,ab OR ‘schizophren*’:ti,ab OR ‘stress*’:ti,ab) OR

(‘optimism’/de OR ‘personal satisfaction’/de OR ‘self concept’/de OR ‘self efficacy’/de OR ‘self-control’/de OR ‘life engag*’:ti,ab OR ‘life satisf*’:ti,ab OR ‘meaning of life’:ti,ab OR ‘purpose in life’:ti,ab OR ‘positive affect*’:ti,ab OR ‘positive emotion*’:ti,ab OR ‘resilien*’:ti,ab OR ‘self concept*’:ti,ab OR ‘self control*’:ti,ab OR ‘self efficac*’:ti,ab OR ‘self esteem*’:ti,ab OR ‘swb’:ti,ab OR ‘well being*’:ti,ab OR ‘wellbeing*’:ti,ab OR ‘Job Satisfaction’:de OR ‘job satisf*’:ti,ab OR ‘work satisf*’:ti,ab OR ‘work engag*’:ti,ab) OR

(‘work capacity evaluation’/de OR ‘effectiveness’:ti,ab OR ‘employabil*’:ti,ab OR ‘presenteeism*’:ti,ab OR ‘productivit*’:ti,ab OR ‘work abilit*’:ti,ab OR ‘work capacit*’:ti,ab OR ‘work disabilit*’:ti,ab OR ‘work function*’:ti,ab OR ‘work participati*’:ti,ab OR ‘work performan*’:ti,ab))

Search terms for Ichushi

("職種別集団"/TH OR "労働衛生"/TH OR “産業保健” OR “事業”/TA OR “産業”/TA OR “企業”/TA OR "ビジネス"/TA OR “仕事”/TA OR "雇用"/TA OR "従業員"/TA OR “被雇用者“/TA OR "雇用者"/TA OR “雇い主”/TA OR “使用者”/TA OR "インフォーマルセクター"/TA OR “非公式経済”/TA OR “非公式部門”/TA OR "インフォーマルワーク"/TA OR "労働者"/TA OR “労務者”/TA OR “作業員”/TA OR “勤労者”/TA OR “スタッフ”/TA OR “職員”/TA OR “社員”/TA OR "職業"/TA OR “稼業”/TA OR “生業”/TA OR "専門家"/TA OR “専門職”/TA OR “有識者”/TA OR "労働力"/TA OR "職場"/TA OR “職域”/TA OR “仕事場”/TA OR "助手"/TA OR "聴覚訓練士"/TA OR "救急"/TA OR “救命”/TA OR “救助”/TA OR "介護士"/TA OR “ヘルパー”/TA OR "臨床医"/TA OR "沿岸警備隊"/TA OR "歯科医"/TA OR “歯医者”/TA OR "刑事"/TA OR “警察”/TA OR “災害対応"/TA OR "医師"/TA OR “医者”/TA OR "在宅医療"/TA OR "在宅ケア"/TA OR “訪問看護”/TA OR "エマージェンシーサービス"/TA OR “緊急サービス”/TA OR “救急サービス”/TA OR "緊急要員"/TA OR “緊急時対応者”/TA OR "救急"/TA OR "消防士"/TA OR "ファーストレスポンダー"/TA OR “第一対応者“/TA OR “初期対応者”/TA OR "総合診療医"/TA OR “かかりつけ医”/TA OR "医療供給者"/TA OR "医療提供者"/TA OR "訪問看護師"/TA OR “訪問看護婦”/TA OR “訪問保健師”/TA OR “訪問保健婦”/TA OR "在宅ケアサービス"/TA OR ("人道"/TA AND (“援助”/TA OR “支援”/TA)) OR "法執行機関"/TA OR “保安官”/TA OR "ライフガード"/TA OR “監視員”/TA OR “救護員”/TA OR “水難救助員”/TA OR "専門医学実習生"/TA OR “研修医”/TA OR “医学生”/TA OR "看護師"/TA OR "看護婦"/TA OR "助産師"/TA OR “助産婦”/TA OR "医療従事者"/TA OR “医療職”/TA OR “コメディカル”/TA OR "警察官"/TA OR "警官"/TA OR "警察職員"/TA OR "女性警察官"/TA OR "婦警"/TA OR "女性警察職員"/TA OR "消防士"/TA OR "消防吏員"/TA OR "消防職員"/TA OR "女性消防士"/TA OR "女性消防吏員"/TA OR “女性消防職員”/TA OR "薬剤師"/TA OR "心理士"/TA OR “心理師” OR "内科医"/TA OR "開業医"/TA OR “弁護士”/TA OR (("救護*"/TA OR "救援"/TA OR “救済”/TA OR “救出”) AND “活動”/TA) OR "レスキュー"/TA OR "セラピスト"/TA OR “療法士”OR "獣医"/TA) AND ("勤務スケジュール許容度"/TH OR "職場内暴力"/TH OR "人間関係"/TH OR "偏見"/TH OR "社会的差別"/TH OR "ハラスメント"/TH OR “リスク調整”/TH OR “リスクマネジメント”/TH OR "組織の文化"/TH OR “組織の方針”/TH OR “リーダーシップ”/TH OR "人員削減"/TH OR "スタッフ開発"/TH OR "従業員の勤務評価"/TH OR "従業員の抗議"/TA OR “実地訓練”/TA OR “実習”/TA OR “実務訓練”/TA OR “現職研修”/TA OR “現場教育”/TA OR "組織介入"/TA OR “参加型介入”/TA OR “チーム介入”/TA OR “仕事のストレス耐性”/TA OR “コンプレストアワー”/TA OR "コンプレストワーク"/TA OR “コンプレストウィーク”/TA OR “日中”/TA OR “フレックス制度”/TA OR “固定時間”/TA OR “フルタイム”/TA OR (“ギグ”/TA AND “エコノミー”/TA) OR “長時間”/TA OR “夜間”/TA OR “夜勤”/TA OR “超過勤務”/TA OR “時間外労働”/TA OR “残業”/TA OR “パートタイム”/TA OR “回復”/TA OR ((“リモート”/TA OR “テレ”/TA) AND “ワーク”/TA) OR “シフト業務”/TA OR ((“セルフ”/TA OR “自己”/TA) AND “スケジューリング”/TA) OR “臨時業務”/TA OR ((“仕事”/TA OR “作業”/TA) AND “スケジュール”/TA) OR “労働時間”/TA OR “勤務時間”/TA OR “交代勤務”/TA OR “ゼロ時”/TA OR “ゼロアワー”/TA OR “予定行動開始時刻”/TA OR ("ライフ"/TA AND “バランス"/TA) OR (“ワーク”/TA AND "ライフ"/TA AND “バランス"/TA) OR ("生活"/TA AND (“家族"/TA OR “家庭”/TA)) OR ("調整"/TA AND (“仕事"/TA OR “作業”/TA)) OR ("生活"/TA AND ("職業"/TA OR “仕事”/TA)) OR “仕事の負担”/TA OR “作業ペース”/TA OR “時間的切迫”/TA OR “裁量の範囲”/TA OR “裁量権”/TA OR “要求度資源”/TA OR “努力報酬”/TA OR “高い要求度”/TA OR “仕事のコントロール”/TA OR “仕事の裁量”/TA OR “仕事の要求度”/TA OR “仕事のストレイン”/TA OR “コントロールの欠如”/TA OR “タスク再構築”/TA OR “タスク管理”/TA OR “低いコントロール”/TA OR “仕事の影響”/TA OR “仕事のストレス”/TA OR “退屈”/TA OR “コーピング”/TA OR “対処”/TA OR ((“コントロール”/TA OR “嫌悪”/TA OR “不快”/TA) AND “仕事”/TA) OR ((“仕事”/TA OR “業務”/TA) AND “内容”/TA) OR “単調”/TA OR “刺激のない”/TA OR “年齢差別”/TA OR “攻撃”/TA OR “侵害”/TA OR “職場内暴力”/TA OR “いじめ”/TA OR “差別”/TA OR “人間関係*”/TA OR “対人関係”/TA OR “ハラスメント”/TA OR “嫌がらせ”/TA OR “同性愛差別”/TA OR “マイクロアグレッション”/TA OR “偏見”/TA OR “人種差別”/TA OR “性差別”/TA OR “静かな職場”/TA OR (“ソーシャル”/TA AND “キャピタル”/TA) OR “社会的資本”/TA OR “孤独”/TA OR “職場での孤独”/TA OR “監督”/TA OR “管理”/TA OR “虐待”/TA OR “職場の葛藤”/TA OR “職場の暴力”/TA OR "リーンマネジメント"/TA OR "リスク調整"/TA OR “リスクマネジメント”/TA OR “安全管理”/TA OR “職場環境”/TA OR “労働条件”/TA OR “作業組織”/TA OR “ワークデザイン”/TA OR “コミュニケーション”/TA OR (“組織”/TA AND “文化”/TA) OR (“組織”/TA AND “機能”/TA) OR “組織の不公正”/TA OR “健康安全”/TA OR (“組織”/TA AND “公正”/TA) OR “組織的正義”/TA OR “リーダーシップ”/TA OR “リーン方式”/TA OR “トヨタ生産方式”/TA OR “労使関係”/TA OR “労働関係”/TA OR “管理業務”/TA OR “管理実施”/TA OR “経営文化”/TA OR “管理尺度”/TA OR “管理策”/TA OR ((“参加”/TA OR “参画”/TA) AND “意思決定”/TA) OR (“組織”/TA AND “方針”/TA) OR “心理社会的リスク”/TA OR “手続き的公正”/TA OR “手続き的不公正”/TA OR “役割明確”/TA OR “技術の自由裁量”/TA OR “役割曖昧さ”/TA OR “役割葛藤”/TA OR “役割不明確”/TA OR “仕事での役割”/TA OR “従業員のパフォーマンス評価”/TA OR “キャリア開発”/TA OR “安定雇用”/TA OR “不安定雇用”/TA OR “オーバースキル”/TA OR “生産性評価”/TA OR “専門能力開発”/TA OR “専門職能開発”/TA OR ((“仕事”/TA OR “職場”/TA) AND “昇進”/TA) OR “スタッフ開発”/TA OR “職員研修”/TA OR “アンダースキル”/TA OR (“チーム”/TA AND “ビルディング”/TA) OR (“チーム”/TA AND “参加”/TA) OR “チームワーク”/TA OR ((“チーム”/TA OR “同僚”/TA OR “同期”/TA ) AND (“仕事”/TA OR “ビルディング”/TA OR “プログラム”/TA))) AND (RD=ランダム化比較試験 OR ((“ランダム化”/TA OR “無作為化”/TA) AND “比較”/TA AND “試験”/TA)) AND (("精神疾患"/TH OR "精神保健"/TH OR "産業心理学"/TH OR "心理的ストレス"/TH OR "適応"/TA OR "気分障害"/TA OR "不安"/TA OR "双極性"/TA OR "バーンアウト"/TA OR "燃え尽き"/TA OR "CMD"/TA OR "抑うつ"/TA OR “鬱”/TA OR "摂食障害"/TA OR "精神疾患"/TA OR “精神障害”/TA OR "精神保健"/TA OR "メンタルヘルス"/TA OR "感情障害"/TA OR "強迫症"/TA OR "ocd"/TA OR "パニック障害"/TA OR "恐怖症"/TA OR "心的外傷後"/TA OR "精神医学的診断"/TA OR "精神科診断"/TA OR "心理障害"/TA OR "心理的ストレス"/TA OR "ptsd"/TA OR "ptss"/TA OR "身体表現性障害"/TA OR "統合失調症"/TA OR "ストレス"/TA) OR ("楽観性"/TH OR "個人的満足"/TH OR "自己概念"/TH OR "自己効力感"/TH OR "セルフコントロール"/TH OR "ライフエンゲイジメント"/TA OR "人生満足"/TA OR “生活満足”/TA OR "人生の意味"/TA OR "人生の目的"/TA OR "ポジティブ感情"/TA OR "レジリエンス"/TA OR “レジリエント”/TA OR "自己概念"/TA OR "セルフコントロール"/TA OR "セルフエフィカシー”/TA OR “自己効力感”/TA OR "セルフエスティーム"/TA OR “自尊心”/TA OR “自尊感情”/TA OR "swb"/TA OR “ウェルビーイング”/TA OR "職務満足度"/TH OR "職務満足度"/TA OR “仕事の満足度”/TA OR "職務満足感"/TA OR ("ワーク"/TA AND ("エンゲイジメント”/TA OR “エンゲージメント”/TA))) OR ("作業能力評価"/TH OR "効率"/TA OR “効果”/TA OR “能率”/TA OR "雇用可能性"/TA OR "プレゼンティズム"/TA OR “疾病就業”/TA OR "生産性評価"/TA OR "職務能力"/TA OR "労働障害"/TA OR "仕事関数"/TA OR “仕事機能”/TA OR "労働参加"/TA OR "パフォーマンス"/TA))
